# CD8 T-cell Recruitment Into the Central Nervous System of Cuprizone-Fed Mice: Relevance to Modeling the Etiology of Multiple Sclerosis

**DOI:** 10.3389/fncel.2020.00043

**Published:** 2020-03-10

**Authors:** Mohammed S. M. Almuslehi, Monokesh K. Sen, Peter J. Shortland, David A. Mahns, Jens R. Coorssen

**Affiliations:** ^1^School of Medicine, Western Sydney University, Penrith, NSW, Australia; ^2^Department of Physiology, College of Veterinary Medicine, Diyala University, Diyala, Iraq; ^3^School of Science, Western Sydney University, Penrith, NSW, Australia; ^4^Department of Health Sciences, Faculty of Applied Health Sciences, St. Catharines, ON, Canada; ^5^Department of Biological Sciences, Faculty of Mathematics and Science, Brock University, St. Catharines, ON, Canada

**Keywords:** castration, gonadally intact, demyelination, gliosis, inside-out, peripheral immune organs, atrophy

## Abstract

Cuprizone (CPZ)-feeding in mice induces atrophy of peripheral immune organs (thymus and spleen) and suppresses T-cell levels, thereby limiting its use as a model for studying the effects of the immune system in demyelinating diseases such as Multiple Sclerosis (MS). To investigate whether castration (*Cx*) can protect the peripheral immune organs from CPZ-induced atrophy and enable T-cell recruitment into the central nervous system (CNS) following a breach of the blood-brain barrier (BBB), three related studies were carried out. In Study 1, *Cx* prevented the dose-dependent reductions (0.1% < 0.2% CPZ) in thymic and splenic weight, size of the thymic medulla and splenic white pulp, and CD4 and CD8 (CD4/8) levels remained comparable to gonadally intact (*Gi*) control males. Importantly, 0.1% and 0.2% CPZ were equipotent at inducing central demyelination and glial activation. In Study 2, combining *Cx* with 0.1% CPZ-feeding and BBB disruption with pertussis toxin (PT) enhanced CD8^+^ T-cell recruitment into the CNS. The increased CD8^+^ T-cell level observed in the parenchyma of the cerebrum, cerebellum, brainstem and spinal cord were confirmed by flow cytometry and western blot analyses of CNS tissue. In Study 3, PT+0.1% CPZ-feeding to *Gi* female mice resulted in similar effects on the peripheral immune organs, CNS demyelination, and gliosis comparable to *Gi* males, indicating that testosterone levels alone were not responsible for the immune response seen in Study 2. The combination of *Cx*+0.1% CPZ-feeding+PT indicates that CPZ-induced demyelination can trigger an *“inside-out”* immune response when the peripheral immune system is spared and may provide a better model to study the initiating events in demyelinating conditions such as MS.

## Introduction

Multiple Sclerosis (MS) is a heterogeneous, inflammatory demyelinating disease of the human central nervous system (CNS) for which the early initiating events and thus underlying etiology remain unclear (Stys et al., 2012; Stys, 2013; Partridge et al., 2015). Currently, there are no effective treatments to prevent disease initiation and progression (Sriram and Steiner, 2005; Vargas and Tyor, 2017). Several animal models such as experimental autoimmune encephalomyelitis (EAE; Glatigny and Bettelli, 2018), Theiler’s murine encephalomyelitis virus (Carrillo-Salinas et al., 2017), and the diphtheria toxin model (Traka et al., 2016) mimic various clinical and pathological features of the disease but no single model replicates the full complexity of disease initiation and progression. Nonetheless, the cuprizone (CPZ) model possesses many key characteristics of MS including demyelination and gliosis (reviewed in Sen et al., 2019b). According to these pathological features, the CPZ model was selected as the most appropriate tool to test the “inside-out” theory of disease initiation (Caprariello et al., 2018). According to this theory, the initiating event in MS is an early, slow degeneration of myelin, which causes the release of potential myelin antigens (as debris) that then activate microglia and macrophages in the presence of proinflammatory mediators. Consequently, myelin antigen presentation by antigen-presenting cells attracts peripheral T- and B-cells into the CNS, triggering a secondary inflammatory reaction that ultimately leads to the progressive autoimmune response characterized clinically (Stys et al., 2012; Stys, 2013).

While there have been numerous reports of CPZ being used to study demyelination, these have not established the involvement of peripheral T-cells at the sites of CPZ-induced demyelination in the CNS (Remington et al., 2007; Partridge et al., 2016; Traka et al., 2016; Tejedor et al., 2017; Sen et al., 2019a). It was thought that as CPZ does not induce a breach of the blood-brain barrier (BBB), T-cells have no access to the CNS (Remington et al., 2007; Skripuletz et al., 2011; Tejedor et al., 2017). However, even when the BBB was breached using pertussis toxin (PT), CPZ evoked marked CNS demyelination, gliosis and changes in the abundance of proteoforms involved in metabolism, immune and synaptic functions, without detectable T-cell infiltration (Sen et al., 2019a). Studies have indicated that the apparent failure to trigger a T-cell-mediated CNS immune response is due to CPZ-induced atrophy of the thymus (the organ responsible for T-cell maturation and differentiation) and spleen (the organ of T and B lymphocyte production; Solti et al., 2015; Martin et al., 2018; Sui et al., 2019; Sen et al., 2019a). Two additional effects of CPZ on the integrity and function of the peripheral immune system include an increase in the abundance of splenic arginase-I (a protein expressed by myeloid-derived suppressor cells in spleen) and a decreased abundance of protein disulfide isomerize (a protein required for assembly of the major histocompatibility complex-I; Partridge et al., 2016). A change in the abundance of these proteins results in suppressed T-cell function (Kang et al., 2009). Likewise, proteoforms of calcium/calmodulin-dependent protein kinase type II subunit-α and leukocyte elastase inhibitor A, involved in T-cell functions, decreased in abundance following CPZ-feeding (Sen et al., 2019a). Moreover, marked changes in the number of mitochondrial proteoforms are suggested to suppress T-cell function (Sen et al., 2019a). Furthermore, a significant reduction in thymic and splenic T-cell numbers, as well as thymocyte apoptosis, was observed after 1–2 weeks of CPZ-feeding (Solti et al., 2015; Martin et al., 2018). Collectively, these studies indicate that the absence of adaptive immune cell involvement in the CNS of CPZ-fed mice is not only due to the intact BBB but is also due to suppression of the adaptive immune system. However, when the BBB was disrupted by PT and the peripheral immune system “boosted” via injection of Complete Freund’s Adjuvant (CFA), an increased pan T-cell marker (CD3^+^) response was observed in the CNS of CPZ-fed mice (Caprariello et al., 2018).

Circulating T-cell numbers are maintained by the thymus gland through various processes including maturation, selection, differentiation and the release of mature T-cells into the blood. During puberty, in both humans and animals, the thymic become atrophied and inactivated (physiological thymic involution) when circulating androgen concentrations increase (Sutherland et al., 2005; Sheean et al., 2015). Notably, experimental testosterone administration (Oner and Ozan, 2002) or CPZ-feeding produced similar effects to natural androgen-induced physiological thymic involution, including thymic atrophy, with the remaining tissue displaying enlarged and degraded mitochondria, lipid droplets and enlarged lysosomes (Solti et al., 2015). Androgen deprivation following castration (*Cx*) increased T-cell levels, thymic function (maturation and differentiation of T-cells) in young and aged mice (Roden et al., 2004; Sheean et al., 2015), and splenic function (T- and B-cells production) in adult mice (Roden et al., 2004). Likewise, *Cx* increased thymic mass (hypertrophy) and resulted in complete restoration of thymus structure (both lymphocytic and epithelial) and function in the primary peripheral immune organs (thymus and bone marrow), including associated T-cell levels (Sutherland et al., 2005). However, no study has investigated whether *Cx*-induced preservation of the thymic and splenic size and function can surmount the suppressive effects of CPZ on the peripheral immune system (i.e., maintain structure and function). Furthermore, no study has tested whether the effect of BBB disruption during CPZ-feeding in female mice, which have naturally low testosterone levels, can lead to T-cell infiltration into the CNS. This is important as MS is seen ~2–3 times more frequently in females than males (Ahlgren et al., 2011; Harbo et al., 2013).

The current work tested the hypothesis that *Cx* protects against the negative effects of CPZ-feeding on thymus and spleen and thus enables T-cell recruitment into the CNS following disruption of the BBB by PT. To investigate this hypothesis, three inter-related studies were carried out using CPZ-feeding in male and female mice. In Study 1, surgical *Cx* was used to protect the adaptive immune system against CPZ effects. In Study 2, *Cx* was combined with 0.1% CPZ-feeding and BBB disruption to test whether the CPZ-induced demyelination initiated an “inside-out” T-cell-mediated response in the CNS. In Study 3, gonadally intact (*Gi*) female mice were fed 0.1% CPZ and the BBB disrupted to test whether females could mount a T-cell response in the CNS, as seen in Study 2. The results suggest a new mouse model for studying the initiating events of MS and further testing the inside-out hypothesis of MS etiology.

## Materials and Methods

### Animals and Monitoring

Weaned (3-week-old) male and/or female C57Bl/6 mice (*n* = 187) were purchased from the Animal Resources Centre, Murdoch, WA, Australia^1^. Mice were acclimatized for 1 week prior to each study and housed (five animals/ventilated GM500 cage, Tecniplast, Buguggiate, VA, Italy) in a controlled environment (12-h light/dark cycle, 50–60% humidity, and 21–23°C room temperature, RT). Standard rodent powder chow (Gordon’s Specialty Stockfeeds, Yanderra, NSW, Australia) and water were available *ad libitum*. Mice were weighed individually at the beginning of the studies, every third day, and prior to sacrifice. Research and animal care procedures were approved by the Western Sydney University animal ethics committee (A11938) in accordance with the Australian Code of Practice for the Care and Use of Animals for Scientific Purposes as laid out by the National Health and Medical Research Council of Australia.

### Bilateral Orchiectomy (Castration, *Cx*)

Adolescent (4-week-old) mice (*n* = 77) were surgically castrated under deep anesthesia using isoflurane (Cenvet, Blacktown, NSW, Australia) 2–3% in 100% oxygen. Mice underwent orchiectomy at this specific age to precede the onset of normal age-related thymic atrophy. The ventral midline of the scrotum was incised (~1 cm) and the tunica exposed. The vas deferens and spermatic artery of each testis were ligated with absorbable polyglycan sutures and the testicles were excised. Then the incision was closed with silk thread (one stitch) and Michel clips (Fine Science Tools, North Vancouver, BC, Canada). Subcutaneous analgesia (Meloxicam 3 mg/kg, Randdolab, NSW, Australia) was injected twice (at the end of surgery and 12 h later) and mice were kept under a heat lamp (~37°C) until awake and mobile. All mice (*Cx* and *Gi*) were inspected daily to identify any abnormal changes in gait, movement, posture, and skin; no abnormalities were observed.

### Cuprizone Administration

**Figure 1:**
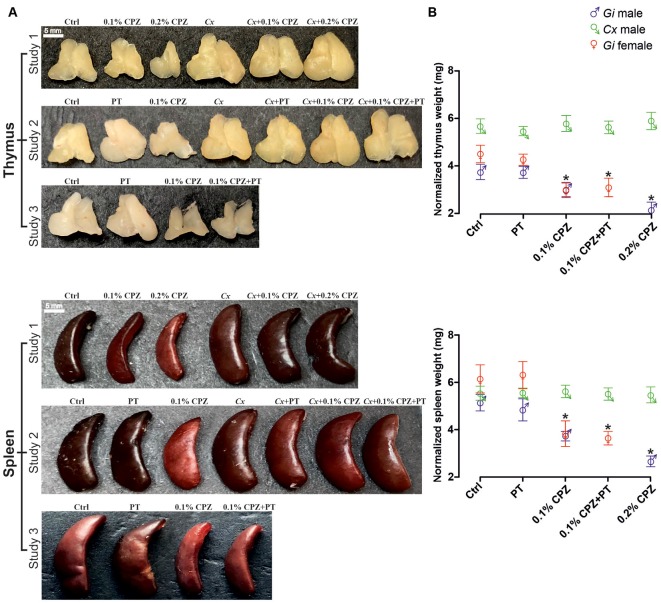
Effects of cuprizone (CPZ)-feeding or castration (*Cx*) on thymus and spleen size and weight. Representative examples of the gross appearance of the thymus and spleen organs **(A)** and quantification of the wet weights **(B)** of the different treatment groups used in the three different studies. In gondally intact (*Gi)* males and females, CPZ-feeding produced a dose-dependent reduction in immune organ mass (*p* < 0.05). In the *Cx* groups, *Cx* significantly increased thymic mass compared to *Gi* males and females (*p* < 0.05) and prevented CPZ-induced thymic and splenic atrophy (*p* > 0.05). In *Gi* female mice CPZ-induced atrophy was indistinguishable to that observed in *Gi* males. Data are presented as mean ± SEM, one-way analysis of variance (ANOVA), *n* = 10 thymic or spleens/group. *Indicates a significant difference from Ctrl (*p* < 0.05).

0.1% or 0.2% CPZ {w/w, [Bis(cyclohexanone)oxaldihydrazone], Sigma–Aldrich, St. Louis, MO, USA\} was used in Study 1. As 0.1% CPZ-feeding induced comparable demyelination and gliosis (Figure 4, Study 1 panel) but less thymic and splenic atrophy than 0.2% CPZ (Figure 1, Study 1 panel), 0.1% CPZ was used in studies 2 and 3. CPZ was mixed with powdered chow to induce demyelination as shown previously (Sen et al., 2019a). Animals were fed either 0.1% or 0.2% CPZ for 2 weeks. Chow was prepared daily with and without CPZ.

**Figure 2:**
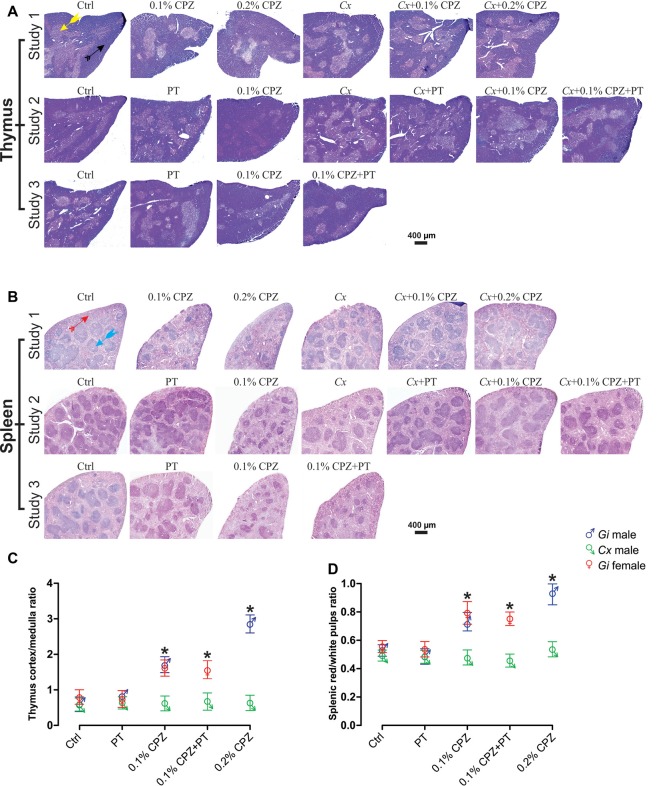
Effects of CPZ-feeding or castration on peripheral immune organs histology. H&E images of the thymus **(A)** and spleen **(B)**, headed arrows identifying the thymic cortex 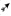), medulla (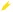), the splenic red pulp (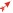) and white pulp (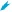) regions, in the different groups of the three separate studies. Quantification of the mean ± SEM thymic cortex/medulla **(C)** and splenic red pulp/white pulp ratios **(D)**. Thymic cortex/medulla and splenic red pulp/white pulp ratios were significantly decreased by 0.1% CPZ in both *Gi* male and female mice and by 0.2% CPZ in *Gi* males compared to Ctrl whereas these ratios were unchanged in *Cx* groups. One-way ANOVA, *n* = 3 thymic or spleens/group, five sections/organ; *indicates significant difference from Ctrl (*p* < 0.05).

**Figure 3:**
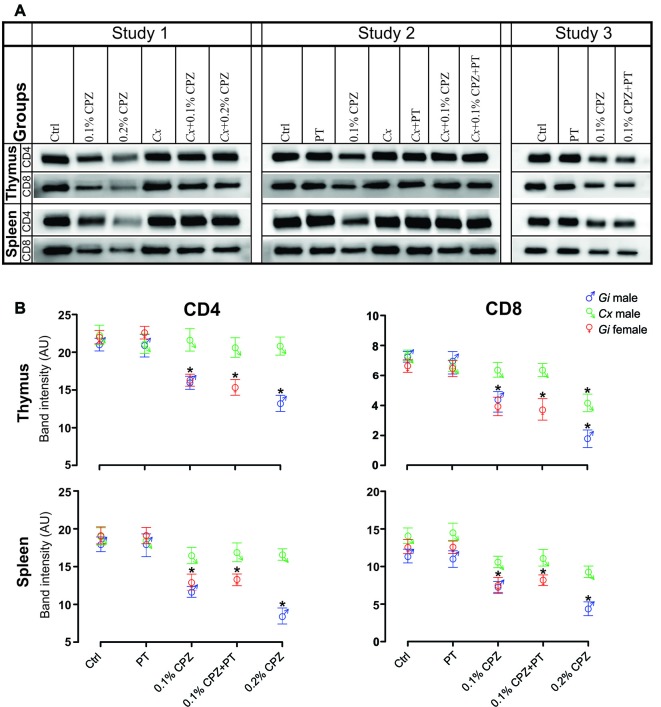
Effects of CPZ-feeding or castration (*Cx*) on CD4 and CD8 signal intensity in the immune organs of male and female mice. Representative examples of western blot images of CD4 and CD8 signal intensities **(A)** and their quantification **(B)** in the different groups in the three separate studies. CD4/8 signal intensities were significantly reduced in the thymus and spleen of *Gi* CPZ-fed male and female mice, whereas CD4/8 signals were completely restored in all *Cx* groups in thymus and spleen except that CD8 signal of the thymus in *Cx*+0.2% CPZ group was markedly attenuated. SDS-PAGE gels were loaded with a 20 μg/well of total protein; one-way ANOVA, *n* = 3 thymic or spleens/group, all samples were processed in triplicate; *indicates significant difference from Ctrl (*p* < 0.05).

**Figure 4:**
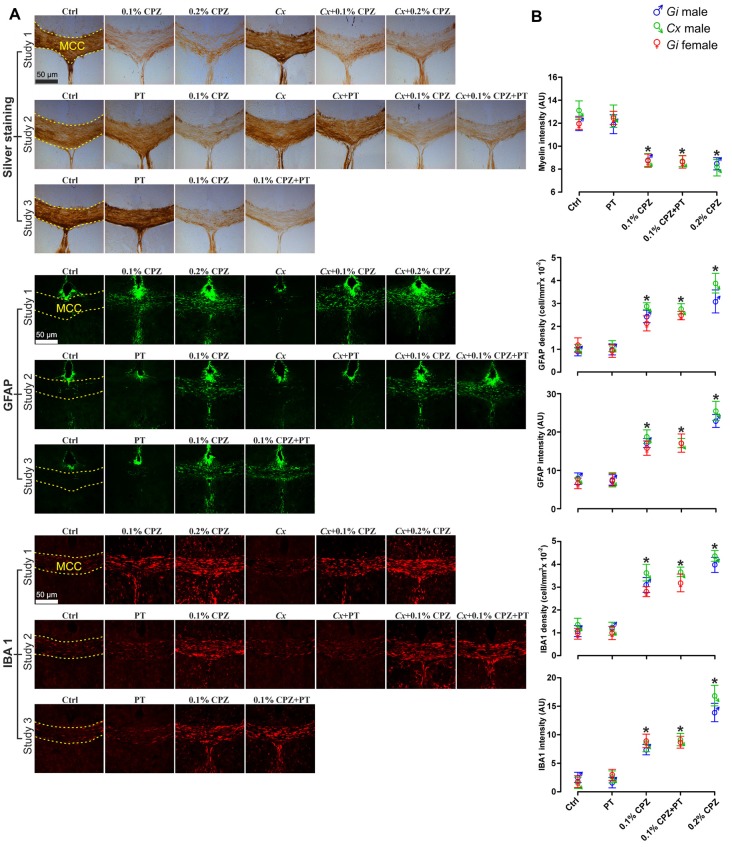
Effects of CPZ-feeding or *Cx* on the central nervous system (CNS) histology. Representative silver, GFAP and IBA 1 staining images **(A)** and quantification **(B)** of silver staining intensity and astrocytes/microglia fluorescence intensity and cell density (cell/mm^3^) in the midline corpus callosum (MCC). 0.1% and 0.2% CPZ-feeding produced identical loss (*p* < 0.05) of myelin intensity in *Gi* male mice. The silver intensity was unaffected following *Cx* in all castrated groups. 0.1% CPZ-feeding to *Gi* male and female mice induced comparable demyelination. Fluorescence intensity and cell density of GFAP and IBA 1 stained sections were significantly (*p* < 0.05) increased in a dose-dependent manner (0.1% < 0.2% CPZ) in *Gi* and *Cx* males. The increase in the fluorescence intensity and density were indistinguishable between males and females when mice were fed with 0.1% CPZ. One-way ANOVA, *n* = 5 mice/group, 10 sections/brain; *indicates significant difference from Ctrl (*p* < 0.05), and dashed yellow line indicates quantification area.

### Pertussis Toxin Injection

In Studies 2 and 3, intraperitoneal injections of PT (Sigma–Aldrich) were used to disrupt the BBB as described previously (Sen et al., 2019a). PT (400 ng/mouse, Sigma–Aldrich) was injected on days 5, 7, and 9 of CPZ-feeding in Studies 2 and 3.

### Experimental Groups

In Study 1, male mice were randomly assigned to one of six groups (*n* = 11/group): controls (Ctrl), 0.1% CPZ and 0.2% CPZ (all *Gi*), *Cx*, *Cx*+0.1% CPZ, and *Cx*+0.2% CPZ. In Study 2, male mice were randomly assigned into one of seven groups: Ctrl, PT, 0.1% CPZ (all *Gi*) and *Cx*, *Cx*+PT, *Cx*+0.1% CPZ and *Cx*+0.1% CPZ+PT (*n* = 11/group). In Study 3, *Gi* female mice (*n* = 11/group) were randomly assigned to one of four experimental groups: Ctrl, PT, 0.1% CPZ and 0.1% CPZ+PT. Mice were assigned to each analysis as follows: three animals/group for western blot analysis, three animals/group for paraffin-embedded tissue staining, five animals/group for free-floating tissue staining.

### Western Blot

Western blot was carried out as previously described (Sen et al., 2019a). Briefly, mice (*n* = 3 animals/group) were euthanized and immediately thereafter brains, spinal cords, thymic and spleens were harvested, washed thoroughly with ice-cold 0.01 M phosphate-buffered saline (PBS, Sigma–Aldrich, St. Louis, MO, USA) containing a protease inhibitor cocktail (4 μM staurosporine, 1 mM α-naphthyl phosphate and 1 mM sodium orthovanadate) to prevent protein degradation during sample preparation and then snap-frozen in liquid nitrogen. Frozen samples were pulverized, solubilized (~1 μl/1 μg tissue) in pre-chilled lysis buffer (25 mM Tris, 1 mM EDTA, 1 mM EGTA, 150 mM NaCl and 1% Triton x-100) and centrifuged at 125,000 *g*, 4°C, for 1 h. Total protein in the supernatant was quantified in each sample using the EZQ protein quantitation kit (Life Technologies, Eugene, OR, USA) with bovine serum albumin (Amresco, Solon, OH, USA) as a calibration standard (Butt and Coorssen, 2005; Churchward et al., 2005). Thymus, spleen, brain, spinal cord and standard purified CD4/8 recombinant proteins (Sino Biological, Wayne, PA, USA) were resolved by 10% sodium dodecyl sulfate-polyacrylamide gel electrophoresis (SDS-PAGE; 100 V for 2 h at 4°C) and then transferred onto polyvinylidene difluoride membrane (PVDF, 0.22 μM pore size, Bio-Rad, Hercules, CA, USA) for 2 h at 4°C; transfer efficiency, determined as previously described (Sen et al., 2019a), was 95.6 ± 1.7% (Supplementary Figures S1A,B). The PVDF blots were incubated at RT in blocking solution 5% skimmed milk (Coles, Hawthorn East, VIC, Australia), in 0.05% Tris-buffered saline-Tween-20, TBST (Amresco) and 1% polyvinylpyrrolidone (Sigma–Aldrich, St. Louis, MO, USA). Membranes were then probed with primary antibodies (Abs) to detect the signal of T-cell subtypes according to their cluster of differentiation (CD)—either anti-CD4 (1:500, Abcam, Cambridge, UK) or anti-CD8—(1:75, Santa-Cruz Biotechnology, Dallas, TX, USA) for 1 h at RT. In the next step, each blot was incubated with a horseradish peroxidase-conjugated (HRP) compatible secondary antibody (CD4 and CD8 blots were incubated with goat anti-rabbit 1:2,000 and mouse anti-mouse, 1:500, respectively) for 1 h at RT. Protein signals were visualized using an enhanced chemiluminescence detection reagent (1 ml/10 cm^2^ for 1 min, Luminata Crescendo Western HRP Substrate, Merck-Millipore, Burlington, MA, USA) and scanned using an ImageQuant^TM^ FUJI LAS-4000 (GE Healthcare, Chicago, IL, USA). The intensity of each protein band was measured using ImageJ software^2^ to calculate the arbitrary value of every single band and the local background was subtracted from these values. The intensity of each band (*n* = 3 bands/mouse, *n* = 3 mice/group) was expressed as a raw value and presented relative to the values obtained from Ctrl mice. The sensitivity of the Abs used in western blot analyses was confirmed by comparing the molecular weight and band size of the signals obtained from the brain, splenic and thymic samples to the signals of the standard purified CD4 or CD8 recombinant proteins.

### Flow Cytometry Analysis

T-cell levels in Study 2 were also analyzed using flow cytometry of thymic, splenic and CNS tissue (*n* = 3 mice/group) to further confirm the western blot and immunohistochemistry results. A standardized application protocol for flow cytometric analysis of T-cell subsets (Miltenyi Biotec)^3^ was applied to prepare a cell suspension suitable for single-cell detection. Briefly, following perfusion with PBS, each organ was placed in a Petri dish and covered with 5 ml of freshly prepared cold (~5°C) PEB buffer (0.5% bovine serum albumin, and 2 mM EDTA dissolved in 0.01 M PBS) and then organs were subjected to mechanical dissociation to obtain a cell suspension. A nylon mesh strainer (70 μm) was used to remove tissue debris and the cell suspension was centrifuged (300 *g*, 10 min, 4°C). Pellets were resuspended in PEB buffer, centrifuged (300 *g*, 10 min, 4°C), and washed with 2 ml 0.01 M PBS before resuspending cells in 1 ml of 0.01 M PBS. Cells (5 μl) were stained with Trypan Blue (Life Technologies, Carlsbad, CA, USA) and counted (Countess automated cell counter, Invitrogen). Each sample was divided into three aliquots: unstained aliquot (negative control) and stained aliquots (CD4 and 8). Cells were stained (~1 × 10^5^ cells/ml) separately with either fluorochrome-conjugated FITC anti-mouse CD4 or PE/Cy7 anti-mouse CD8a antibodies (1:50 dilution, BioLegend, San Diego, CA, USA) for 1 h in the dark at RT while shaking. After adding 2 ml of 0.01 M PBS and centrifugation (300 *g*, 10 min, 4°C) the pellet was resuspended in 100 μl of 0.01 M PBS and analyzed using a MACSQuant^®^ Flow Cytometer (Bergisch Gladbach, NRW, Germany). A total of 20,000 events were analyzed for each sample. The lymphocyte gate was defined by forward and side scatters and the CD4^+^ and CD8^+^ T-lymphocytes gates were defined by FITC and PE channels, respectively. Data were analyzed on FlowJo software (version 10, LLC, Ashland, OR, USA) and presented as the absolute number of dot blots/quadrant 1 (Q1) or 3 (Q3) for CD4 and CD8 T-cell populations, respectively and the ratio of CD4 to CD8 counts.

### Histology and Immunohistochemistry

#### Tissue Preparation

Mice (*n* = 5/group in each study) were deeply anesthetized with isoflurane (2–3% in 100% oxygen) and perfused transcardially with cold 0.9% saline followed by 4% paraformaldehyde (in 0.1 M phosphate buffer, Sigma–Aldrich, St. Louis, MO, USA). Samples (CNS, thymic and spleens) were collected from the different groups and postfixed using 4% paraformaldehyde at 4°C for 3 days, then kept in 0.02% sodium azide (prepared in 0.01 M PBS, Sigma–Aldrich, St. Louis, MO, USA) at 4°C until processed for staining within 1 month. Whole CNS was immersed in a 30% sucrose solution for 48 h at RT until it sank, confirming cryo-protection. Tissue was then embedded in 4% gelatine (Chem-Supply, Gillman, SA, Australia) in cryomolds (Sakura, Torrance, CA, USA). Gelatine embedded tissue was then embedded in Tissue-Tek^TM^ optimal cutting temperature compound (Sakura) and sectioned with a cryostat (Leica, Wetzlar, HE, Germany) at −20°C. Serial coronal sections of the CNS (40 μm) were either transferred to 6-well plates containing 0.01 M PBS (free-floating for immunohistochemistry) or mounted onto 0.5% gelatine-coated slides for silver staining. Sections were placed in an anti-freezing solution (glycerol, ethylene glycol and 0.01 M PBS at 1:1:2 by volume) at −20°C until stained (≥1 month), as described previously (Sen et al., 2019a). To prepare tissue for hematoxylin and eosin (H&E) staining, thymic and spleens were postfixed in 10% formalin for 3 days and then dehydrated in ethanol (70%, 80%, 95%, and 100%) for 2 h each, cleared using xylene for 2 h and embedded in paraffin (SLEE medical GmbH, Mainz, Germany). Organs were placed in embedding cassettes (Sigma–Aldrich) and kept at 4°C to form solid paraffin blocks. Then tissue blocks were sectioned (10 μm) using a manual rotary microtome (Leica Microsystem, Wetzlar, Germany). A consecutive series of tissue sections were mounted onto glass gelatine coated slides and dried at RT for 1 h. Excess paraffin wax was removed from the slides by heating them at 55°C for 30 min).

#### Silver Myelin Staining and Quantification

Staining and quantification were performed as previously described (Sen et al., 2019a). Briefly, slide-mounted brain sections were air-dried for 48 h at RT and then immersed in 10% formalin (Sigma–Aldrich) for 14 days. Slides were incubated with a 2:1 solution of pyridine (VWR, Radnor, PA, USA) and acetic anhydride (Merck, Darmstadt, HE, Germany) followed by incubation with freshly prepared ammoniacal silver nitrate (Chem-Supply) for 45 min for staining. Mounting medium (DPX, Merck) and coverslips (Knittel Glass, Braunschweig, NI, Germany) were used to cover the sections and left to dry for 72 h at RT. All brain sections were imaged with bright field Olympus Carl Zeiss microscope (Zeiss, Jena, TH, Germany) using the same settings (i.e., exposure time, magnification and illumination intensity). Images were analyzed by ImageJ software as follows: for each section, the region of interest (midline corpus callosum, MCC) was contoured and the mean optical density was measured as mean gray value [i.e., summation of all the pixels in the region of interest divided by the number of pixels, with individual pixels ranging from black (0) to white (256)]. The results were plotted as the reciprocal of the light intensity to measure the amount of myelin. The anatomical landmark was identified as described previously (Paxinos and Franklin, 2012; Sen et al., 2019a).

#### Immunofluorescence Staining and Quantification

Staining and quantification were carried out as previously described (Sen et al., 2019a). In short, non-specific binding was blocked using 10% goat serum (Sigma–Aldrich) for 2 h at RT and sections were incubated overnight at RT with primary Abs: glial fibrillary acidic protein (anti-GFAP-AlexaFluor 488, 1:1,000, Merck-Millipore, Burlington, MA, USA), ionized calcium-binding adaptor molecule 1 (rabbit anti-IBA 1, 1:1,000, Wako, Japan), anti-CD4 (rabbit anti-CD4, 1:200, Abcam), or anti-CD8 (mouse anti-CD8, 1:100, Santa-Cruz Biotechnology, Dallas, TX, USA), then washed thrice with 0.01 M PBS. Sections were then incubated with secondary corresponding Abs [either goat anti-rabbit IgG, 1:500, Invitrogen (USA) or mouse anti-mouse IgGκ, 1:100 (Santa-Cruz Biotechnology)] for 2 h at RT and washed 3 × 10 min with 0.01 M PBS. Sections were counterstained with 1.5 mg/ml of nuclear counter-stain Vectashield plus 4′,6-diamidino-2-phenylindole (DAPI, Vector Laboratories, Burlingame, CA, USA) and imaged using a fluorescence Olympus Carl Zeiss microscope (Zeiss, Germany) using the same exposure time and magnification settings. Fluorescence intensity measurement of GFAP and IBA 1 stained sections was performed with ImageJ software as described above. Positively stained cells with GFAP and IBA 1 (and co-stained with DAPI) were counted using unbiased stereo investigator optical fractionator workflow software (see Figure 4 and Supplementary Stereology Excel File) as described previously (Sen et al., 2019a). However, due to the low counts per unit area and regional heterogeneity of CD8^+^ cell distribution; these cells were counted manually across the entirety of each section in the cerebrum, cerebellum, brainstem and spinal cord (three animals/group, 10 sections/brain or spinal cord, five sections/cerebellum or brainstem). In order to confirm that the CD8^+^ expressing cells were a distinct population (i.e., from microglia) brain and spinal cords sections were double-labeled with CD8 and IBA 1 antibodies and co-stained with DAPI.

#### Assessment of BBB Integrity

To assess the permeability of the BBB following PT injection in Study 2, peroxidase-based immunohistochemistry was used to visualize immunoglobulin G (IgG) in the CNS. In Study 3, the same method was used to breach BBB and the results were consistent with Study 2 (*data not shown*). Free-floating coronal sections were washed as described above and non-specific binding and endogenous peroxidase activity were blocked using peroxidase blocking solution (Dako, Carpinteria, CA, USA). Sections were then incubated for 1 h at RT in biotinylated anti-mouse IgG secondary Ab (1:100 dilution, Vector Laboratories Inc., Burlingame, CA, USA). Sections were then incubated with avidin-biotin-peroxidase complex (1:200 dilution, ABC complex, Vector Laboratories) for 30 min at RT, and visualized using diaminobenzidine (DAB, Vector Laboratories Inc., Burlingame, CA, USA) solution (0.05% DAB in 0.005% H_2_O_2_). All incubation and reaction steps were performed in parallel for all sections, at RT, to ensure that the DAB staining was comparable (i.e., equal number of sections from each experimental group exposed to the same batch of DAB stain and incubated for the same amount of time). Sections were then mounted on slides and cover-slipped using DPX mounting medium and left to dry overnight. Sections were imaged by bright field Olympus Carl Zeiss microscope (Zeiss, Germany) and quantified using ImageJ software to measure the IgG color intensity as described above.

#### Haematoxylin and Eosin (H&E) Staining

Staining with H&E was performed in thymus and spleen tissue to assess the structural changes associated with CPZ-feeding. Following removal of excess paraffin from the tissue (see “Tissue Preparation” section), slides were rinsed in xylene for 10 min (to remove the remaining paraffin wax) and rehydrated by dipping in a series of alcohol washes (100%, 80%, 50% and 0%, five times each solution). In the next step, hematoxylin (4 g/l, Merck-Millipore, Burlington, MA, USA) was used for 3 min to stain the sections followed by washing with tap water. Slides were then dipped into acid alcohol for 30 s to differentiate the cytoplasm and transferred into Scott’s Bluing solution to stain the nucleus. Then the tissue was stained with eosin (0.5%) to counterstain acidic components of cells (e.g., cytoplasmic proteins) and washed with tap water followed by ascending concentrations of alcohol (70%, 95%, and 100%) to dehydrate the tissue and xylene to clear it. Slides were covered with mounting medium (Merck) and sealed with coverslips (Cardiff et al., 2014). Tissue sections were imaged with an Olympus Carl Zeiss bright field microscopy. ImageJ software was used to analyze various histomorphometric measurements of the thymus and spleen as previously described (Tryphonas et al., 2004). Briefly, the total area (mm^2^) of each thymus section (three mice/group, five sections/mouse) was contoured and measured. All regions of the thymic medulla in each section were also contoured individually to obtain the area of each region; these values were summed to yield the total medullary size of each thymic section. The total section area was then subtracted from the medulla area to get the total thymic cortex area. Averages of cortical and medullary values of each mouse in all groups were calculated. Finally, the average values of cortex were divided by average medullary values to obtain the thymic cortex/medulla ratio. The same strategy was applied to spleen sections in order to measure the splenic red pulp/white pulp ratio.

### Statistical Analysis and Graphing

All data were presented as mean ± standard error of the mean (SEM). Bodyweight data was analyzed using the two-way analysis of variance (ANOVA). All other data were analyzed using a one-way ANOVA and individual differences were determined using Newman–Keuls *post hoc* multiple comparison analyses. Statistically significant differences compared to Ctrl were considered when *p* < 0.05 and indicated by asterisks (*) in all graphs. Statistical analyses and graphing were performed using GraphPad Prism 7.03 software^4^ (San Diego, CA, USA). Figures were assembled using the CorelDRAW graphics design software version 2019^5^ (Ottawa, ON, Canada).

## Results

### Study 1: Castration Counteracts the Peripheral Effects of CPZ

To quantify the impact of CPZ on the thymus and spleen, and to test whether *Cx* could protect these organs against the effects of CPZ-feeding, prepubescent male C57Bl/6 mice (i.e., 4 weeks old) were surgically castrated and subsequently fed with powdered chow containing 0.1% or 0.2% CPZ for 2 weeks, in parallel with Ctrl mice. Weight gain in all *Cx* males (*Cx*, *Cx*+0.1% CPZ, and *Cx*+0.2% CPZ groups) was significantly reduced compared to *Gi* CPZ-fed males and *Gi* Ctrl during the first 3 days of CPZ-feeding and this effect continued to the end of the study. During the second week of CPZ-feeding, significant reductions in body weight gain were observed in the CPZ-fed animals compared to healthy Ctrls (Supplementary Figure S2). By the end of the experiment, the weight differences between Ctrl mice and that fed 0.1% or 0.2% CPZ were approximately 9% and 15%, respectively. This rank order was preserved when comparing *Cx* alone and 0.1% or 0.2% CPZ *Cx* animals, in which the weight differences were approximately 8% and 15% respectively. Following 2 weeks of 0.1% or 0.2% CPZ-feeding, the size (Figure 1A) and weight of the thymus and spleen (normalized to body weight, Figure 1B, Study 1 panels) in *Gi* males was reduced (*p* < 0.05) in a dose-dependent manner (thymus: 13.3 ± 0.03% vs. 28.4 ± 0.03%, spleen: 27.6 ± 0.04% vs. 42.4 ± 0.04% for 0.1% vs. 0.2% CPZ) compared to their respective Ctrls. In contrast, thymic mass was increased by 69.0 ± 4.1%, (*p* < 0.05) following *Cx* compared to *Gi* Ctrls at 2 weeks and this effect remained despite CPZ-feeding. While the splenic mass of *Cx* mice was not different from *Gi* Ctrls, *Cx* did prevent the CPZ-induced reduction in splenic weight seen in the *Gi* group (Figure 1B).

### Castration Restored the Histological Architecture of Thymus and Spleen

In *Gi* male mice, CPZ-feeding induced dose-dependent reductions in medulla area (0.2% CPZ: 18.5 ± 1.0 > 0.1% CPZ: 9.6 ± 1.0 mm^2^) without any change in cortical area (Figures 2A,C, Table 1) resulting in a dose-dependent increase in the thymic cortex/medulla ratio (0.2% CPZ: 1 ± 0.03 > 0.1% CPZ: 0.7 ± 0.04 > Ctrl: 0.5 ± 0.03). In contrast, the thymic cortex/medulla ratio was unchanged by feeding 0.1% or 0.2% CPZ to *Cx* mice (Table 1). Similarly, H&E staining confirmed that feeding CPZ to *Gi* mice induced a dose-dependent (0.1% < 0.2% CPZ) reduction in splenic red pulp/white pulp ratio [Figures 2B,D; i.e., the white pulp size was affected by CPZ-feeding (Table 1)]. In contrast, CPZ-feeding had no effect on white pulp area following *Cx*, i.e., white pulp size of *Cx*+0.1% and *Cx*+0.2% CPZ groups were indistinguishable (*p* < 0.05) from *Gi* Ctrl spleens. Taken together, these results showed that *Cx* protected mice against the deleterious effects of CPZ-feeding on the spleen and thymus regardless of CPZ dose.

**Table 1:** Histomorphometric evaluation of thymus and spleens from *Gi* and *Cx* males, and *Gi* female mice, exposed to CPZ.

	Group	Thymic cortex	Thymic medulla		Splenic red pulp	Splenic white pulp	
Study 1	Ctrl	33.8 ± 3.3	40.1 ± 2.6		7.7 ± 0.9	14.5 ± 1.4
	0.1% CPZ	32.6 ± 3.7	18.5 ± 1	↓	6.7 ± 0.5	9.5 ± 1.3	↓
	0.2% CPZ	27.1 ± 3	9.6 ± 1.1	↓	5.7 ± 0.6	5.9 ± 0.4	↓
	*Cx*	33.1 ± 4.3	56.6 ± 2.4	↑	8.1 ± 0.6	15.3 ± 1.5
	*Cx*+0.1% CPZ	32.2 ± 0.8	51.7 ± 2.5	↑	7.9 ± 0.3	14.3 ± 1.1
	*Cx*+0.2% CPZ	31.6 ± 3.8	49.8 ± 4.3	↑	7.8 ± 0.4	14.5 ± 0.5
Study 2	Ctrl	33.8 ± 3.3	40.14 ± 2.57		7.7 ± 0.9	14.5 ± 1.5
	PT	35.0 ± 3.9	39.37 ± 4.23		7.0 ± 1.1	13.9 ± 0.6
	0.1% CPZ	32.6 ± 3.7	18.45 ± 0.97	↓	7.4 ± 0.5	8.5 ± 0.7	↓
	*Cx*	33.1 ± 4.3	56.62 ± 2.41	↑	8.1 ± 0.6	15.3 ± 1.5
	*Cx*+PT	31.6 ± 4.3	44.94 ± 4.44	↑	8.4 ± 0.4	14.7 ± 0.5
	*Cx*+0.1% CPZ	32.2 ± 0.8	51.67 ± 2.46	↑	7.9 ± 0.3	14.3 ± 1.1
	*Cx*+0.1% CPZ+PT	32.4 ± 0.7	53.49 ± 6.39	↑	8.3 ± 0.2	15.9 ± 2.3
Study 3	Ctrl	32.3 ± 3.4	41.19 ± 3.1		8.2 ± 0.8	15.2 ± 1
	PT	28.8 ± 3.1	39.11 ± 4.2		8.4 ± 0.2	15.9 ± 1.5
	0.1% CPZ	28.1 ± 1.8	17.39 ± 0.8	↓	7.9 ± 0.5	10.2 ± 1.4	↓
	0.1% CPZ+PT	26.8 ± 1.5	17.29 ± 1.1	↓	7.6 ± 0.2	10.1 ± 0.6	↓

*Values are presented as mean area (mm^2^) ± SEM (*n* = 3 thymic or spleens/group, five histological sections/organ). Statistically significant changes are indicated as higher* (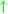) *or lower* (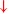) *relative to Ctrl when p < 0.05*.

### Castration Preserved CD4/8 Signal in the Thymus and Spleen Against the Impact of CPZ

Western blot analysis indicated a significant dose-dependent reduction (0.2% > 0.1% CPZ) in the signal intensity of CD4 and CD8 T-cells in both thymus and spleen of *Gi* CPZ males compared to *Gi* Ctrl males (Figures 3A,B). The effect of 0.1% or 0.2% CPZ on CD4 signals in both thymus and spleen was prevented by *Cx* despite feeding with CPZ. Moreover, the CD8 signal was indistinguishable from the Ctrl group when *Cx* combined with 0.1% or 0.2% CPZ in spleen but only with the *Cx*+0.1% CPZ group in the thymus. Although the effect of CPZ on thymic CD8 signal in the *Cx*+0.2% CPZ group was ameliorated by *Cx* (compared with the *Gi* male at the same dose), CD8 signal was significantly (*p* < 0.05) decreased in the *Cx*+0.2% CPZ group compared to the Ctrl group (Figure 3B). Overall, 0.2% CPZ had a significant (*p* < 0.05) suppressive impact on the levels of CD4/8 in both thymus and spleen compared to 0.1% CPZ in *Gi* mice. *Cx* induced preservation of thymic and splenic CD4/8 levels in both *Cx*+0.1% CPZ and *Cx*+0.2% CPZ groups compared to *Gi* Ctrl males.

### CPZ-Induced Demyelination

Silver staining revealed that 0.1% and 0.2% CPZ-feeding induced a significant decrease in MCC myelin of *Gi* male mice compared to *Gi* Ctrl males (Figures 4A,B, Study 1 panel) and the extent of demyelination was indistinguishable between 0.1% or 0.2% CPZ. *Cx* alone, or when combined with 0.1% or 0.2% CPZ, did not affect the myelin staining intensity compared to *Gi* Ctrl males. Overall, 0.1% CPZ-feeding was as effective at producing demyelination as 0.2% CPZ in mice, with or without *Cx*.

### Effects of CPZ and *Cx* on Glial Activation

GFAP and IBA 1 staining were used to quantify astrocytes and microglia, respectively, in the MCC. Following CPZ-feeding in *Gi* males, cell density (cell/mm^3^) and fluorescence intensity of both GFAP and IBA 1 were significantly increased in a dose-dependent manner (0.2% > 0.1% CPZ) compared to *Gi* Ctrl (Figures 4A,B, Study 1 panels, GFAP, IBA 1). Combining *Cx* with 0.1% or 0.2% CPZ-feeding did not change the cell density or the fluorescence intensity of either GFAP or IBA 1 staining. Both 0.1% and 0.2% CPZ-feeding induced a strong glial response in the demyelinated areas in the MCC.

### Study 2

**Figure 5:**
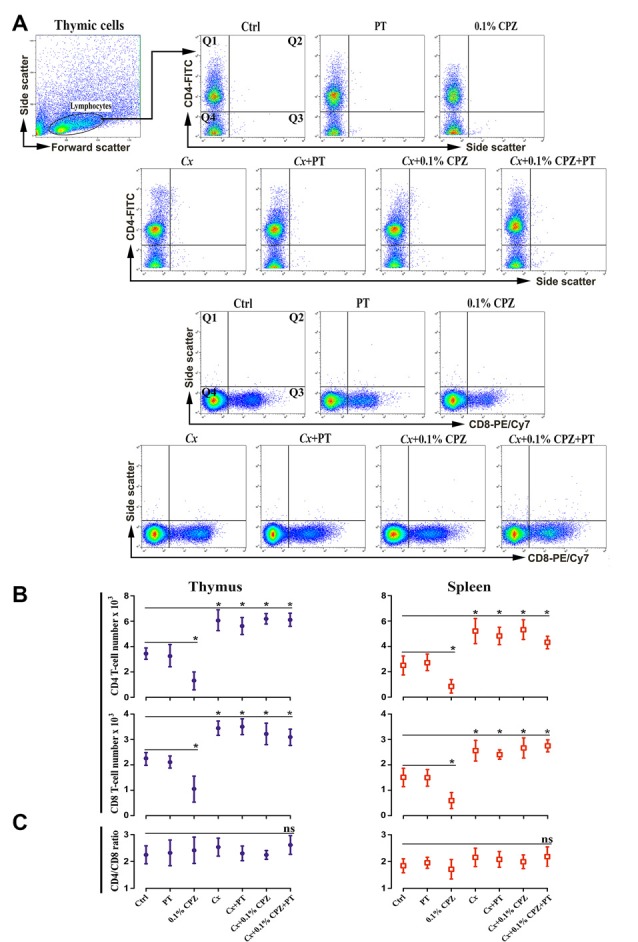
Flow cytometric analysis of T-cell subpopulations (CD4^+^ and CD8^+^) in the immune organs of male mice. Representative flow cytometry dot blots of thymic CD4 and CD8 T-cells **(A)** and the quantification of their cell number in thymus and spleen tissue from Study 2 **(B)**. CD4/8 number was significantly reduced in the thymus and spleen of 0.1% CPZ-fed mice compared to Ctrl. The numbers of CD4/8 increased in all *Cx* groups compared to Ctrl and were unaffected by CPZ. Cell counts in the quadrant 1 (Q1) for CD4 and quadrant 3 (Q3) for CD8 were used to quantify cell number in each experimental group. No significant (ns, *p* > 0.05) changes in the ratio of CD4/CD8 were observed in thymus and spleen **(C)**. A one-way ANOVA (*n* = 3 thymic or spleens per group, 20,000 events/sample) was used to determine significant differences from Ctrl (*p < 0.05).

Intraperitoneal injections of PT in male mice had no synergistic effect when combined with 0.1% CPZ in both the *Gi* and *Cx* males. As seen in Study 1, CPZ-feeding resulted in comparable reductions in weight gain (Supplementary Figure S2, Study 2) and a reduction in thymic and splenic mass (Figures 1A,B, Study 2 panels). *Cx* increased the thymic weight but the splenic mass remained unchanged compared to *Gi* Ctrl. Western blot analysis showed significant reductions (*p* < 0.05) in CD4/8 signals in *Gi* animals fed 0.1% CPZ compared to Ctrl whereas, in *Cx* groups, CD4/8 signals remained unchanged when fed 0.1% CPZ (Figures 3A,B, Study 2 panels). Likewise, flow cytometric analysis of thymocytes and splenocytes showed significant decreases (*p* < 0.05) in CD4/8 T-cell subpopulations in the 0.1% CPZ group compared to Ctrl, whereas in the *Cx* groups CD4/8 T-cell subpopulations were significantly increased (*p* < 0.05, Figures 5A,B). In addition, there were no changes in the ratio of CD4/CD8 across all groups (*p* > 0.05, Figure 5C). Myelin, GFAP and IBA 1 intensities in the PT group of *Gi* males were indistinguishable from Ctrl. PT injection did not change the amount of demyelination nor the extent of microglia and astrocyte activation when combined with *Cx*, 0.1% CPZ, or *Cx*+0.1% CPZ (Figures 4A,B, Study 2 panels). The PT-mediated breach of the BBB was confirmed by a significant increase in the intensity of IgG staining in the hippocampal region of the PT injected groups (Supplementary Figures S3A,B). Quantification was performed in the hippocampus because the intensity of the stain was highest there relative to regions (Supplementary Figure S3).

### CD8^+^ T-cell Number Increased in the CNS Following PT Injection in *Cx*-CPZ-Fed Mice

**Figure 6:**
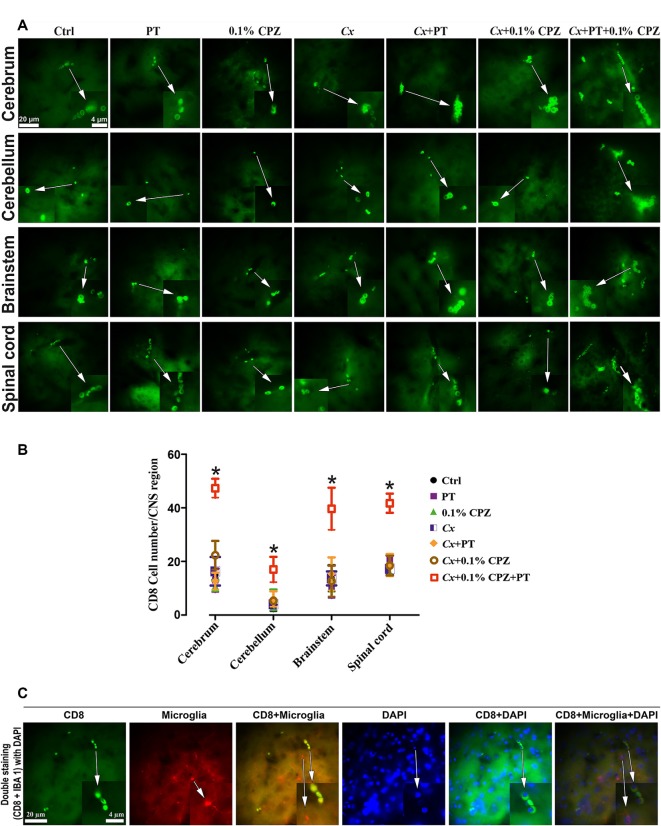
Combined effects of CPZ-feeding and *Cx* with pertussis toxin (PT) on CD8^+^ T-cells in the CNS. Representative images of CD8^+^ T-cells detected in the CNS tissue **(A)** and quantification **(B)** of CD8^+^ T-cell numbers in the CNS (cerebrum, cerebellum, brainstem, and spinal cord). In Study 2, the number of CD8^+^ T cells was significantly (*p* < 0.05) higher in each part of the CNS in the *Cx*+0.1% CPZ+PT group than in all other groups. Section **(C)** shows representative images of double labeling with CD8 and IBA 1 antibodies in the brain tissue, nuclear DAPI staining, and merged images confirming the identity of CD8^+^ T-cells. As indicated by white arrows, regions of the images were magnified five times and these appear as insets at the bottom of the image panel; one-way ANOVA, *n* = 3 mice/group, 10 sections/brain or spinal cord, five sections/cerebellum or brainstem; *indicates significant difference from Ctrl (*p* < 0.05).

In a previous study, CD4 and/or CD8 were not detected following PT injection in the CNS of *Gi* male mice fed 0.1% CPZ (Sen et al., 2019a). In contrast, when 2 weeks of 0.1% CPZ-feeding was combined with *Cx* and PT treatment (*Cx*+0.1% CPZ+PT, Figures 6A,B) CD8^+^ cell numbers in the CNS increased. The distribution of CD8^+^ T-cells in the stained slices was not homogeneous, with CD8^+^ cells appearing either as individual cells or in small groups of cells distributed widely throughout the CNS (cerebrum, cerebellum, brainstem, and spinal cord). In *Gi* and *Cx* animals, CD8^+^ cells were rarely encountered and their numbers were unaffected by CPZ-feeding (range between 0–23 cells/section) compared to Ctrl. When CPZ-feeding and *Cx* were combined with PT, the number of CD8^+^ cells increased (<2-fold) in multiple regions of the brain (whole parenchyma) and spinal cord (most notably in the gray matter and around the central canal, Figures 6A,B). Double labeling of CNS sections with CD8 and IBA 1 antibodies (and co-stained with nuclear DAPI staining) confirmed that CD8 and IBA 1 were expressed in distinct cell populations (Figure 6C).

**Figure 7:**
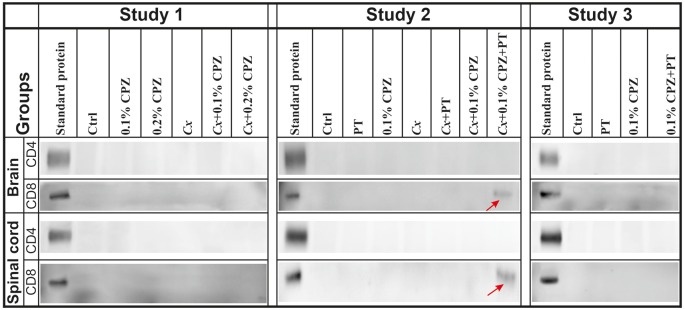
Detection of CD4/8 signals in the CNS homogenate. Representative images of western blot analysis of CD4 and CD8 antigen detection in the brain and spinal cord. No CD4 signal was detected in the CNS tissue across all groups in Studies 1–3. In contrast, the CD8 signal band was detected only in brains and spinal cords of *Cx*+0.1% CPZ+PT mice in Study 2 (red arrows). SDS-PAGE gels were loaded with 60 μg/well of total protein, all samples were processed in triplicate, and standard purified CD4 and CD8 protein concentrations were 5 ng/well and 5 μg/well, respectively.

In addition, using the same western blot analysis of the brain and spinal cord homogenate samples (three independent 60 μg sample loads per animal, three animals/group) we replicated the previous finding that neither CD4 nor CD8 signals were detected in the CNS homogenates of Ctrl, 0.1% CPZ and PT treated *Gi* males (Sen et al., 2019a). However, in the *Cx*+0.1% CPZ+PT mice, CD8 signal was detected in the CNS homogenates, whereas the CD4 signal was not (Figure 7, Study 2). Furthermore, neither CD4 nor CD8 signals were detected in CNS homogenates in either Study 1 or Study 3 (Figure 7, Studies 1 and 3).

**Figure 8:**
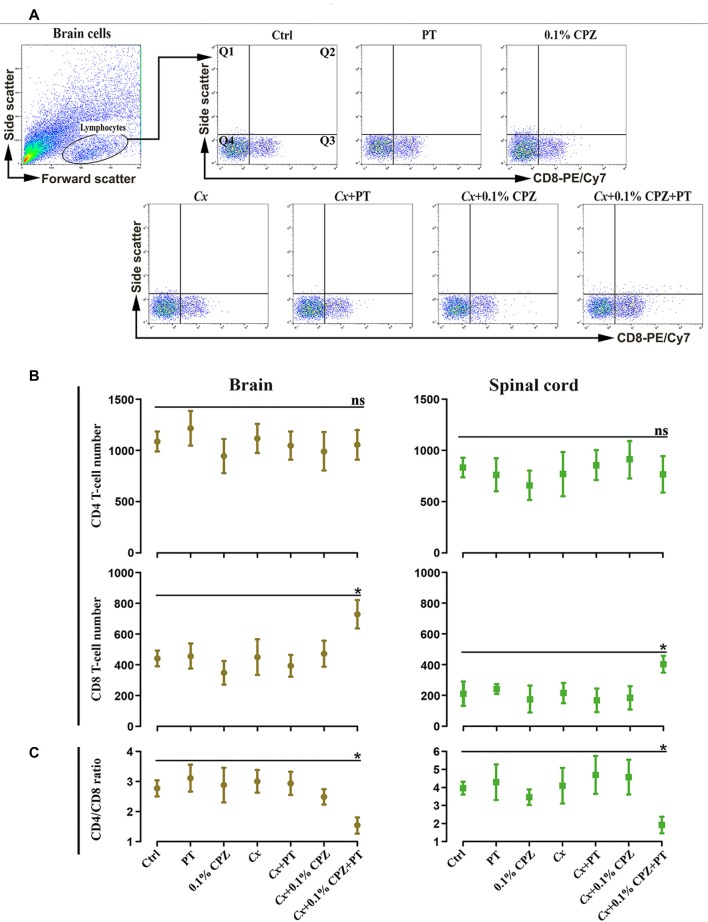
Flow cytometric analysis of T-cell subpopulations (CD4 and CD8) in the brain and spinal cord of male mice. Representative CD8^+^ T-cells dot blots in the brain **(A)** and quantification **(B)** of CD4^+^ and CD8^+^ T-cell numbers in the brain and spinal cord. In Study 2, no significant (ns) changes in the number of CD4^+^ T-cell of the brain and spinal cord were observed, whereas the number of CD8^+^ T-cells was significantly (*p* < 0.05) increased only in the brains and spinal cord of the *Cx*+0.1% CPZ+PT group. Cell counts in quadrant 3 (Q3) for CD8 were used to quantify cell number in each experimental group. The ratio of CD4/CD8 in the brain and spinal cord was unchanged (ns) in all groups except in the *Cx*+0.1% CPZ+PT group in which the ratio was significantly decreased in both brain and spinal cord **(C)**. A one-way ANOVA (*n* = 3 thymic or spleens per group, 20,000 events/sample) was used to determine significant differences from Ctrl (*p < 0.05).

The increased CD8^+^ T-cell signal intensity (western blot) and number (immunohistochemistry) in the brain and spinal cord tissue of the *Cx*+0.1% CPZ+PT mice were also confirmed using flow cytometric analysis (Figures 8A,B). Moreover, CD4^+^ T-cells count via flow cytometry did not show any changes among all groups (i.e., CD4 cell number in Ctrls: brain 1,087 ± 314; spinal cord 834 ± 176). The ratio of CD4/CD8 was unchanged in all groups compared to Ctrl except in the *Cx*+0.1% CPZ+PT group, in which the ratio was significantly decreased (*p* < 0.05, Figure 8C).

### Study 3

BBB disruption was applied in Study 3 to test whether CD8^+^ T-cells could be increased in the CNS of *Gi* female mice as observed in *Cx*+0.1% CPZ+PT treated males. Feeding 0.1% CPZ to *Gi* female mice led to reduced body weight gain (Supplementary Figure S2), severe thymic and splenic atrophy (Figures 1A,B) and changes in histological architecture (thymic cortex/medulla and splenic red pulp/white pulp ratios). These effects were indistinguishable from those observed in *Gi* males (Figures 2A,B). Notably, the reductions in CD4/8 were comparable to those in *Gi* males (Figures 3A,B). Likewise, the levels of demyelination, and microglia and astrocyte activation were indistinguishable from those observed in age (and treatment) matched *Gi* and *Cx* males (Figures 4A,B). In contrast to the *Cx* males, following 0.1% CPZ+PT, CD4/8 T-cells were not detected in CNS tissue of *Gi* female mice (Figure 6).

## Discussion

To better understand why T-cells have been so infrequently found at sites of demyelination in the CPZ animal model, the impact of CPZ-feeding on the peripheral immune organs that are responsible for the maturation, differentiation and production T-cells, namely the thymus and spleen, was assessed (Cesta, 2006; Pearse, 2006). While previous studies have shown CPZ-induced thymic and splenic atrophy, they did not attempt to alleviate these effects (Solti et al., 2015; Martin et al., 2018; Sui et al., 2019; Sen et al., 2019a). The morphological and histopathological changes to the thymus following CPZ-feeding observed here and in other studies (Solti et al., 2015), are remarkably similar to those observed and attributed to androgen-dependent thymic involution that can be reversed by androgen depletion (Roden et al., 2004; Tang et al., 2012). Consistent with this observation, Study 1 demonstrated that *Cx* reversed the 0.1% or 0.2% CPZ-induced atrophy of the thymus and spleen and preserved CD4/8 signal intensity in both immune organs. In Study 2, *Cx*, when combined with 2 weeks of CPZ-feeding and disruption of the BBB (using PT), resulted in demyelination, gliosis and CD8^+^ T-cell infiltration in the cerebrum, cerebellum, brainstem, and spinal cord. In female mice (Study 3), combining CPZ-feeding with BBB disruption resulted in CNS demyelination and gliosis, but no CD8^+^ T-cell infiltration into the brain or spinal cord. Taken together, these results indicate that CPZ-induced demyelination in the CNS can trigger a CD8^+^ T-cell-mediated central response when the peripheral immune system is preserved.

As previously documented, CPZ-feeding led to a significant reduction in weight gain (Franco-Pons et al., 2007; Torkildsen et al., 2009; Ye et al., 2013; Chang et al., 2017; Sen et al., 2019a) and a reduction in thymic and splenic weights (Solti et al., 2015; Martin et al., 2018; Sui et al., 2019; Sen et al., 2019a). Although *Cx* was timed to precede the onset of normal age-related thymic atrophy (Sheean et al., 2015), *Cx* did not prevent the CPZ-induced suppression of total body weight gain. However, *Cx* did counteract the CPZ-induced suppression of thymus size and weight, spleen weights, and changes in the thymic medulla and splenic white pulp (structures responsible for T-cell development and production). Furthermore, western blot analysis confirmed that *Cx* countered all CPZ-induced suppression of CD4/8 signal in the thymus and spleen. In the absence of the protective effects of *Cx*, the direct effects of CPZ-feeding on the thymus and spleen provide a plausible explanation as to why prior studies did not show the involvement of T-cells in the CNS of CPZ-fed mice (Remington et al., 2007; Tejedor et al., 2017; Sen et al., 2019a). Likewise, the demonstration that CPZ ameliorates and/or prevents the clinical and pathological features of EAE and Theiler’s virus encephalomyelitis (Maña et al., 2009; Herder et al., 2012), is consistent with CPZ suppressing peripheral immune function and hence improving both clinical and pathological features driven by T-cells. Based on this, and prior work (Solti et al., 2015; Martin et al., 2018; Sui et al., 2019; Sen et al., 2019a), it is clear that suppressive effects of CPZ on the thymus and spleen, and thus T-cells (CD4^+^ and CD8^+^ lymphocytes), are not conducive to study effects mediated by peripheral immune cells *unless* strategies to protect and/or boost the peripheral immune system are employed.

Broadly speaking, the toxic effects of CPZ have been attributed to copper chelation that interferes with cellular and mitochondrial metabolism and results in the formation of mega- or enlarged-mitochondria in the liver and thymus of mice (Suzuki, 1969; Hoppel and Tandler, 1973; Solti et al., 2015). Likewise, in the mitochondria of oligodendrocytes, increased reactive oxygen species and decreased activity of respiratory chain complexes resulted from CPZ-feeding (Gudi et al., 2014; Faizi et al., 2016). Additionally, in the atrophied thymus, enlarged mitochondria, myelin bodies, enlarged lysosomes, and lipid droplets were observed following CPZ-feeding (Solti et al., 2015). The enlarged lysosomes are the result of increased membrane permeability, and the release of lysosomal proteases activates caspases, ultimately leading to thymocyte apoptosis (Zhang et al., 2009; Veto et al., 2010).

T-helper (CD4^+^) cells require copper to transcribe interleukin-2 (Hopkins and Failla, 1999)-a process that may be suppressed due to the copper chelating action of CPZ (Emerson et al., 2001). These effects of CPZ on mitochondrial function and thymocyte apoptosis may explain why western blot and flow cytometry analyses of the thymus from CPZ-fed *Gi* mice identified a reduction in CD4/8 T-cell levels whereas *Cx* counteracted this effect, at least in part due to the hypertrophy resulting from the ablation of androgens. Supporting this, it has been suggested that androgen depletion may be involved in the regulation of mitochondrial dysfunction and toxic responses (Liu et al., 2019).

The suppressive effects of androgens on the structure and function of the thymus have been extensively investigated. The most prominent effects are mitochondrial dysfunction-induced thymocyte apoptosis and thymic atrophy, which result in a subsequent reduction of circulating T-cells (Olsen et al., 1994, 1998; Veto et al., 2010). However, *Cx* countered these effects. The preservation of thymic CD4/8 signal intensity in *Cx* groups, in the presence of CPZ, may not be limited to the effects of *Cx* on developing thymic cortical and medullary cells, but include effects on bone marrow-derived stem cells. *Cx* increased the number of thymocyte precursor cells in the bone marrow and enhanced their differentiation into mature thymocytes in the thymus, leading to thymic regeneration (Sutherland et al., 2005). These effects of *Cx* are consistent with other findings in young and aged animals in which orchiectomy induces rapid restoration of thymopoiesis, a significant increase in the proliferation of immature thymocytes, and a reduction in apoptosis (Olsen et al., 1994; Roden et al., 2004; Page et al., 2006; Sheean et al., 2015). In the current work, combining a 0.1% CPZ with *Cx* meant that the suppressive effects of CPZ on the peripheral immune system were counter-balanced by ablating the normal androgen-mediated involution of the thymus, thereby preserving peripheral immune function.

Immature T-cells are selected in the thymic tissue according to their specificity to T-cell receptors (e.g., CD4^+^, CD8^+^, and forkhead P3^+^ regulatory T-cells), to develop functional and self-tolerant T-cell repertoires (positive selection), and induce a central tolerance to eliminate of autoreactive T-cells (negative selection). The maturation of thymocytes occurs in the thymic cortex, whereas the differentiation and negative selection of T-cells occurs in the thymic medulla (Klein et al., 2014; Kurd and Robey, 2016). This suggests that the CPZ-mediated effects in the medulla (i.e., on maturation, differentiation, and selection of T lymphocytes) might decrease the capacity of the thymus to support a T-cell-mediated immune response in the CNS. In contrast, in the *Cx*-CPZ-fed animals, the increased thymic mass, particularly in the medulla, is still capable of initiating/sustaining a T-cell response through maturation, differentiation and selection processes.

The central role of androgen-mediated thymic atrophy has been suggested on the basis of the presence of androgen receptors on the thymic epithelial cells (Savino and Dardenne, 2000) and developing thymocytes (Viselli et al., 1995) that are stimulated by circulating androgens, thus resulting in thymic atrophy (Olsen et al., 2001). CD4 and CD8 T-cells are the major cell subsets that express androgen receptors (Sutherland et al., 2005), and are the major cell subsets that are reduced by CPZ-feeding. Therefore, the combined effects of androgen-dependent involution and CPZ-feeding in the *Gi* male mice resulted in a reduction in thymic mass and function, whereas in CPZ-fed *Cx* mice, thymic mass (and CD4^+^ and CD8^+^ thymocytes) are preserved.

As in the thymus, CPZ-feeding reduced splenic wet weight, the size of its white pulp (a site of B and T-cell production) and CD4/8 T-cell levels, effects that have been attributed to CPZ-induced mitochondrial dysfunction and oxidative stress (Martin et al., 2018). Furthermore, a highly sensitive top-down proteomic analysis of spleens from CPZ-fed *Gi* male mice identified a significant increase in arginase-I abundance as well as decreased levels of protein disulfide isomerase (Partridge et al., 2016). The increased level of arginase-I, expressed by myeloid-derived suppressor cells, may have contributed to the decreased T-cell levels in the CPZ-fed mice. Furthermore, protein disulfide isomerase is required for the appropriate assembly of major histocompatibility complex-I (Kang et al., 2009). A reduction in major histocompatibility complex-I molecules and consequent reduction in antigen presentation will impair cytotoxic T-cell (CD8^+^) activity and thus limit subsequent T-cell activation and clonal expansion, thereby providing another mechanism by which T-cell activity in CPZ-fed mice was compromised. Following orchiectomy, spleens were enlarged with increased white pulp areas, an effect attributed to the mitotic expansion of splenic lymphoid cells capable of responding to immune stimuli (Dean et al., 1984). This increased mitotic division of splenic lymphoid cells in *Cx* groups may compensate for the loss of mass and cell number induced by CPZ-feeding.

In Studies 1 and 2, 2 weeks of CPZ-feeding led to extensive demyelination that was unaffected by androgen ablation in *Cx* mice. However, it has been shown that androgen depletion by *Cx* increased the severity of demyelination after 5 weeks of CPZ-feeding (Patel et al., 2013). The authors attributed this to the lack of protective androgen effects in the CNS following *Cx*. This suggests that at this early time point (i.e., 2 weeks of CPZ-feeding) lack of androgens does not have any deleterious effects on CNS demyelination. Androgens work as trophic factors to maintain the development and the plasticity of neuronal tissue regulates glial cell activity and enhances myelination (Melcangi et al., 2001; Garcia-Segura and Melcangi, 2006). These protective effects of androgens are sustained by androgen receptors that are found on all cell types in the CNS including astrocytes, microglia, oligodendrocytes, and neurons (Jung-Testas and Baulieu, 1998; Garcia-Ovejero et al., 2002). In contrast, others have argued that testosterone administration increases oligodendrocytosis by amplifying the toxic damage through α-amino-3-hydroxy-5-methyl-4-isoxazolepropionic acid (AMPA)/Kainate receptor activation (Marin-Husstege et al., 2004; Cerghet et al., 2006). Other studies have shown that as little as 2 weeks of CPZ-feeding can induce significant demyelination in the mouse CNS (Pfeifenbring et al., 2015; Caprariello et al., 2018). In the current work, short term (2 weeks) feeding of 0.1% CPZ produced comparable demyelination and gliosis to the standard 0.2% CPZ dose; yet 0.1% CPZ has a more limited effect on the peripheral immune structures. These findings are consistent with previous data showing that 0.1% CPZ-feeding was as effective at producing demyelination as 0.2% CPZ but with less impact on the spleen (Sen et al., 2019a).

Having demonstrated that *Cx* did not enhance CPZ-induced demyelination and gliosis, yet protected against the suppression of peripheral immune organs, Study 2 showed that combining *Cx* and CPZ-feeding with disruption of the BBB resulted in a CD8^+^ T-cell immune response in the CNS. The disruption of the BBB was confirmed in Studies 2 and 3 by an increased presence of IgG in the brain parenchyma following PT injection. It has been argued that the disruption of the BBB increases the possibility of adaptive immune cells traversing the barrier leading to activation and recruitment of T-cells due to myelin antigen presentation by microglia following oligodendrocytosis and the degeneration of myelin induced by CPZ (Caprariello et al., 2018). Disruption of the BBB alone, even when combined with either CPZ-feeding or castration, was not sufficient to initiate a CD8^+^ T-cell mediated response in the CNS. This means that the release of myelin antigens following CPZ-feeding is the key step to induce a peripheral immune response and provides strong evidence for the inside-out theory of MS disease initiation (Stys et al., 2012; Stys, 2013). The increase in the number of CD8^+^ T-cells observed in the CNS using immunohistochemistry was further confirmed by flow cytometric analysis of brain and spinal cord tissue. Notably, the CD8 signal was also detected in brain and spinal cord tissue by western blot analysis when *Cx* was combined with 0.1% CPZ and PT. This preferential increase in CD8 levels was not observed in previous CPZ-feeding studies that did not include strategies to protect the peripheral immune system (Remington et al., 2007; Partridge et al., 2016; Traka et al., 2016; Tejedor et al., 2017; Sen et al., 2019a) or which the focus of analysis was on a pan T-cell marker (CD3; Caprariello et al., 2018). While it has been argued that “cellular sources of CD8 were reactive macrophages/microglia” (Zhang et al., 2009), here, double labeling of the brain and spinal cord sections with IBA 1 and CD8 showed distinct microglia and CD8 cell populations. Notably, as observed in human MS lesions (del Pilar Martin et al., 2008), most of the CD8^+^ T-cells were observed in the CNS parenchyma surrounding the blood capillaries, suggesting recent infiltration of these cells into the CNS tissue. Previous studies showed that *Cx* significantly increases peripheral CD8^+^ T-cell numbers and function in humans and mice (Page et al., 2006; Tang et al., 2012). The exact mechanism of CD8 T-cell infiltration was not examined in this study but seems most likely to have occurred following BBB disruption with subsequent expansion in response to the CPZ-induced demyelination, consistent with the inside-out theory of MS (Stys et al., 2012; Stys, 2013).

Indeed, the process of infiltration may have been facilitated by the activation of innate immune cells (microglia and astrocytes), their presentation of myelin antigens and the release of tumor necrosis factor-γ and reactive oxygen species (Bonetto et al., 2017), that are known to increase the permeability of the BBB and attract CD8^+^ T-cell (Suidan et al., 2006). Notably, the predominance of CD8^+^ T-cell in the CNS of mice (Study 2) resembles that seen at MS lesion sites where the key steps include recruitment and clonal expansion (Hauser et al., 1986; Friese and Fugger, 2005), with CD8^+^ outnumbering CD4^+^ T-cells by 3–10 fold (Booss et al., 1983; Babbe et al., 2000). In contrast, CD4^+^ and CD8^+^ T-cell levels in the blood of MS patients are comparable to those observed in healthy individuals (Waschbisch et al., 2014), suggesting that the initiation of immune involvement in MS patients is a result of CD8^+^ T-cell expansion at the site of the lesion (Crawford et al., 2004).

The inability to detect the CD4 antigen by western blot in the CNS did not appear to be due to failure of the techniques used as CD4 antigens were readily identified in the spleen, thymus, and in samples of brain tissue that were spiked with a recombinant CD4 protein standard (Sen et al., 2019a). Notably, the specificity of the CD4 antibody has been confirmed by others (Forlani et al., 2019; Zhao et al., 2019), and has been used to detect CD4^+^ cells in the spinal cord of EAE animals (Sen et al., 2019a). In addition to the copper chelating actions of CPZ causing apoptosis of T-helper cells, the lack of CD4 detection in the CNS may, at least in part, be due to a PT-mediated inhibition of CD4 chemokine receptors (G_i_ protein-coupled receptors), which play a key role in migration and extravazation of CD4^+^ T cell into target tissues (Su et al., 2001; Alt et al., 2002). Conversely, a PT-mediated inhibition of chemokine receptors that protect against apoptotic signaling in CD4^+^ T-cells may also contribute to the absence of CD4^+^ T-cells (Vlahakis et al., 2002; Rot and von Andrian, 2004). Although EAE (a widely used animal model of MS) has provided important insights into how CD4^+^ T-cell responds to peripherally injected myelin antigens (Steinman and Shoenfeld, 2014), it does not address the role of central (e.g., *inside-out*), triggers of immune responses (Caprariello et al., 2018). Therefore, the EAE model does not replicate the same circumstances of disease *initiation* as seen in humans: namely, a response to a slow, long-term endogenous demyelination in the CNS, a predominance of brain and spinal cord pathology (vs predominance of spinal cord pathology), and CD8 (vs. CD4) mediated T-cell involvement (Wiendl and Hohlfeld, 2002). In contrast, here, Study 2 resulted in a pathological pattern that included focal demyelination *inside* the CNS and a subsequent *outside* infiltration of CD8^+^ T-cell into the CNS, closely resembling the pattern observed in type III MS lesion in humans that is characterized by severe oligodendrocytosis and T-cell infiltration (presence of T-lymphocytes, macrophages and large numbers of oligodendrocytes loss; Lucchinetti et al., 2000).

The hypothesis underlying Study 3 was that combining CPZ-feeding with BBB disruption in females that have naturally lower testosterone levels, would result in CD8^+^ T-cell infiltration into the CNS similar to that seen in *Cx* males (Study 2). However, the preservation of thymic and splenic mass (and their associated CD4^+^ and CD8^+^ thymocytes) seen in *Cx* males was not reproduced in *Gi* females. This indicates that the preservation of immunological function observed following *Cx* in males cannot be solely ascribed to the loss of androgen production but may be attributed to other gonadal hormones shared by males and females e.g., activin and inhibin (Licona et al., 2006). Indeed, testosterone production offers some protection against the development of immunological disorders in males, such as MS, an effect that may explain why the disease occurs more frequently (2–3-fold) in females (Compston and Coles, 2008; Orton et al., 2010; Wallin et al., 2012). The high incidence of the disease in females has been ascribed to the rapid response of their immune system to any immunological stimulus such as vaccination because the concentrations of serum immunoglobulin are higher than in males (Azar et al., 2017; Trend et al., 2018). Aspinall and Andrew reported that age-related involution of the thymus (thymic atrophy) occurred earlier in *Gi* male mice than in females due to the effects of androgens. Consequently, the numbers of CD4^+^ and CD8^+^ thymocytes were reduced prior to positive selection (Aspinall and Andrew, 2001). Additionally, CPZ-feeding to female mice induced a 2–3-fold reduction in the weights of their uterus and ovaries compared to Ctrls (Taylor et al., 2010). This means that androgens in normal female mice have a slower effect on age-related thymus atrophy than in males. In addition, CPZ-feeding effects on the uterus and ovaries may reduce the levels of circulating androgens in females, thereby somewhat alleviating their effects on peripheral immune organs.

In Study 3, it was found that feeding CPZ to prepubescent female mice induced similar effects on the CNS (demyelination and gliosis) and peripheral immune organs as seen in males, consistent with previous studies (Taylor et al., 2010; Martin et al., 2018). This indicates that there are no sex differences with regard to the deleterious effects of CPZ-feeding in juvenile or adult mice.

Overall, this work has addressed two important questions relevant to the etiology of MS. First, could demyelination and activation of innate immunity trigger an autoimmune response similar to that seen in MS? Second, might MS primarily be initiated by demyelination that then triggers peripheral T-cell recruitment to the CNS lesions? Concerning the first question, the data indicate that the peripheral actions of CPZ are an impediment to studying the role of the peripheral immune system in response to central demyelination, despite damage to the CNS that is quite reminiscent of MS. With regard to the second question, the data confirm that, subsequent to CPZ-induced demyelination, CD8^+^ T-cells are recruited to the CNS following disruption of the BBB when the peripheral immune system is intact. The fact that peripheral CD3^+^ T-cells are recruited into the CNS following CPZ-feeding and BBB disruption (with PT), when combined with strategies that protect or *“boost”* the peripheral immune system, i.e., peripheral CFA injections (Caprariello et al., 2018), provides further support for an “*inside-out”* initiation of MS.

Despite using multiple techniques (organ weight, western blot analysis, immunohistochemistry and flow cytometry) to confirm the interaction (and its reversal by *Cx*) between CPZ-feeding, peripheral immune structures and CD4/8 T-cell recruitment to the CNS, future activation and proliferation assays are required to determine the impact of individual or combined (*Cx*+0.1% CPZ+PT) treatments on T-cell function and their capacity to traverse the BBB. This need for detailed functional studies is reinforced by other work showing that the use of CFA injection to “boost” the peripheral immune system resulted in an enhanced infiltration of cells expressing the pan T-cell marker CD3 following CPZ-induced demyelination (Caprariello et al., 2018).

## Conclusion

The findings fully establish that feeding juvenile male mice with 0.1% or 0.2% CPZ for 2 weeks produced equivalent levels of demyelination and gliosis in the corpus callosum. However, 0.1% CPZ-induced less thymic and splenic atrophy, histopathology and a more modest effect on T-cell levels than the standard CPZ dose (0.2%). Moreover, the suppression of peripheral immune structures by CPZ explains why the recruitment of peripheral immune cells into the CNS has not been reported in previous studies. Castration protected against CPZ-induced thymic and splenic atrophy and thus the loss of CD4/8 T-cells. Furthermore, the addition of PT to castrated CPZ-fed mice resulted in CD8^+^ T lymphocyte infiltration into CNS parenchyma, providing strong supporting evidence for the “inside-out” hypothesis in the etiology of MS. Demonstration of CD8^+^ T-cell recruitment into the CNS of CPZ-fed mice, albeit castrated male mice, provides a potential new variant of the CPZ model with which to explore the early events involved in CNS demyelinating diseases like MS and thus move beyond the focus on glia responses and pan T-cell markers in the literature. However, while 0.1% CPZ-feeding in *Gi* female mice induced peripheral immune organ atrophy, T-cell signal suppression, and CNS demyelination and gliosis identical to that seen in males, BBB disruption did not result in T-cell infiltration into the CNS, indicating that testosterone levels alone are not responsible for the central CD8 T-cell response.

## Data Availability Statement

All datasets generated for this study are included in the article/Supplementary Material.

## Ethics Statement

The animal study was reviewed and approved by Western Sydney University animal ethics committee (A11938) in accordance with the Australian Code of Practice for the Care and Use of Animals for Scientific Purposes as laid out by the National Health and Medical Research Council of Australia.

## Author Contributions

MA, MS, PS, DM, and JC conceived the study and provided all resources. MA and MS carried out the lab work in collaboration. MA analyzed the data and drafted the original manuscript. All authors reviewed the different versions of the manuscript and approved the final version.

## Conflict of Interest

The authors declare that the research was conducted in the absence of any commercial or financial relationships that could be construed as a potential conflict of interest.

## Footnotes

^1^http://www.arc.wa.gov.au

^2^https://imagej.nih.gov/ij/

^3^http://www.miltenyibiotec.com

^4^www.graphpad.com

^5^www.coreldraw.com
