# Supplementary material for: CD8 T-cell Recruitment Into the Central Nervous System of Cuprizone-Fed Mice: Relevance to Modeling the Etiology of Multiple Sclerosis
Source: Front Cell Neurosci. 2020 Mar 10;14:43. doi: 10.3389/fncel.2020.00043 (PMC7076139; doi:10.3389/fncel.2020.00043)
Supplement: Supplementary file 1 [file Data_Sheet_1.PDF]

CD8 T-cell recruitment into the central nervous system of cuprizone-fed mice: Relevance to modelling the aetiology of Multiple Sclerosis

Mohammed SM Almuslehi[1,2], Monokesh K Sen[1], Peter J Shortland[3] David A Mahns*[1] and Jens R Coorssen*[4]

[1]School of Medicine, Western Sydney University, Locked Bag 1797, Penrith, NSW 2751, Australia.

[2]Department of Physiology, College of Veterinary Medicine, Diyala University, Diyala, Iraq.

[3]School of Science and Health, Western Sydney University, Locked Bag 1797, Penrith, NSW 2751, Australia.

[4]Department of Health Sciences, Faculty of Applied Health Sciences, and Department of Biological Sciences, Faculty of Mathematics and Science, Brock University, Ontario, Canada.

*Co-corresponding authors

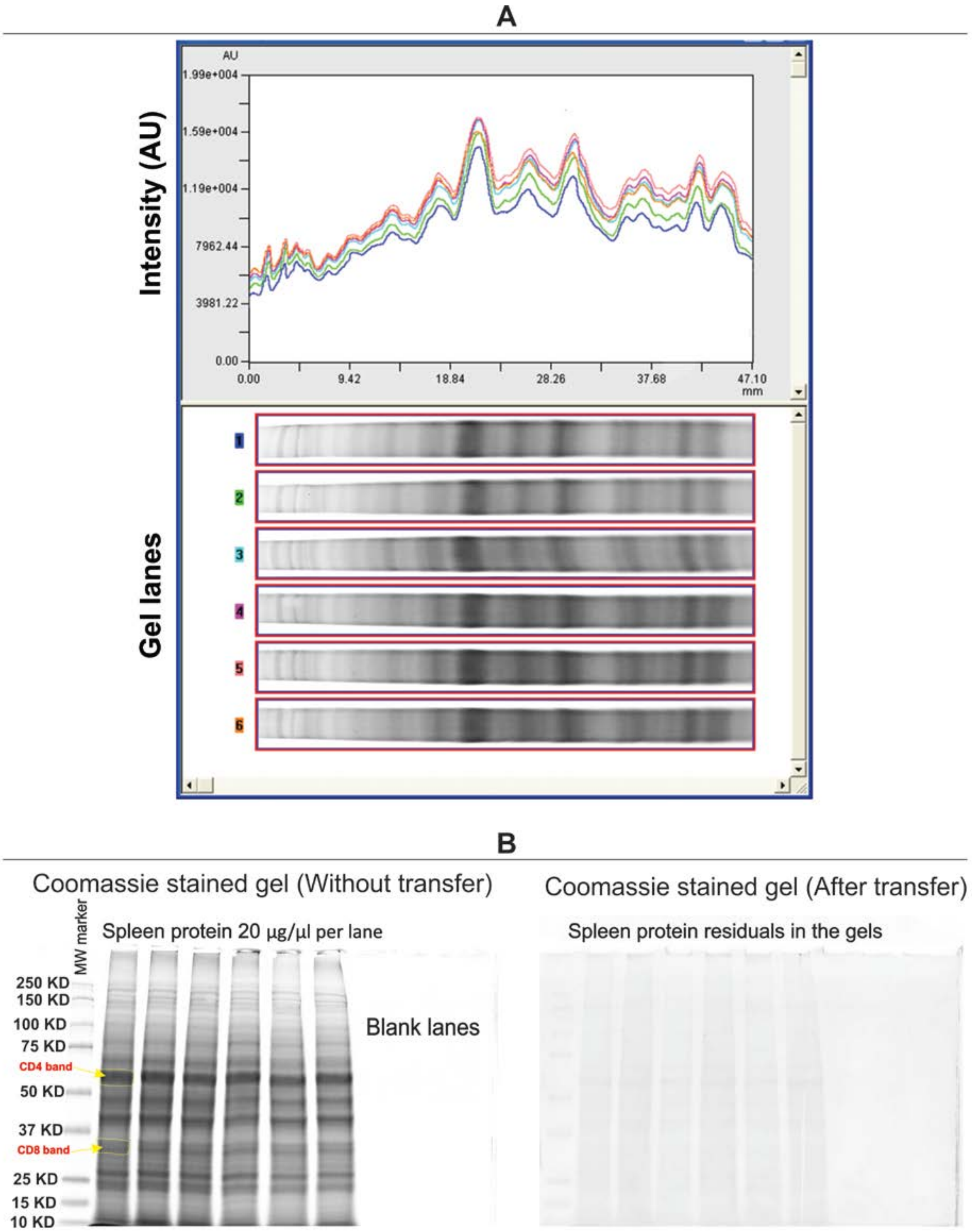

Supplementary FIGURE 1: Protein transfer efficiency and protein loading

Representative Multi Gauge line scan of the intensity (AU) of Coomassie Brilliant Blue stained proteins (without transfer) showing the consistency of the protein loads across the gel lanes (A). Line scans for each lane are colour coded. Images of SDS-PAGE gels that were processed in parallel and stained with Coomassie Brilliant Blue, left image (gel without transfer) and right side image (gel after transfer), to quantify the efficiency of protein transfer from the gel onto PVDF membrane (B). Protein bands intensities were equal across all bands and lanes and the protein transferred efficiently (95.6±1.7%).

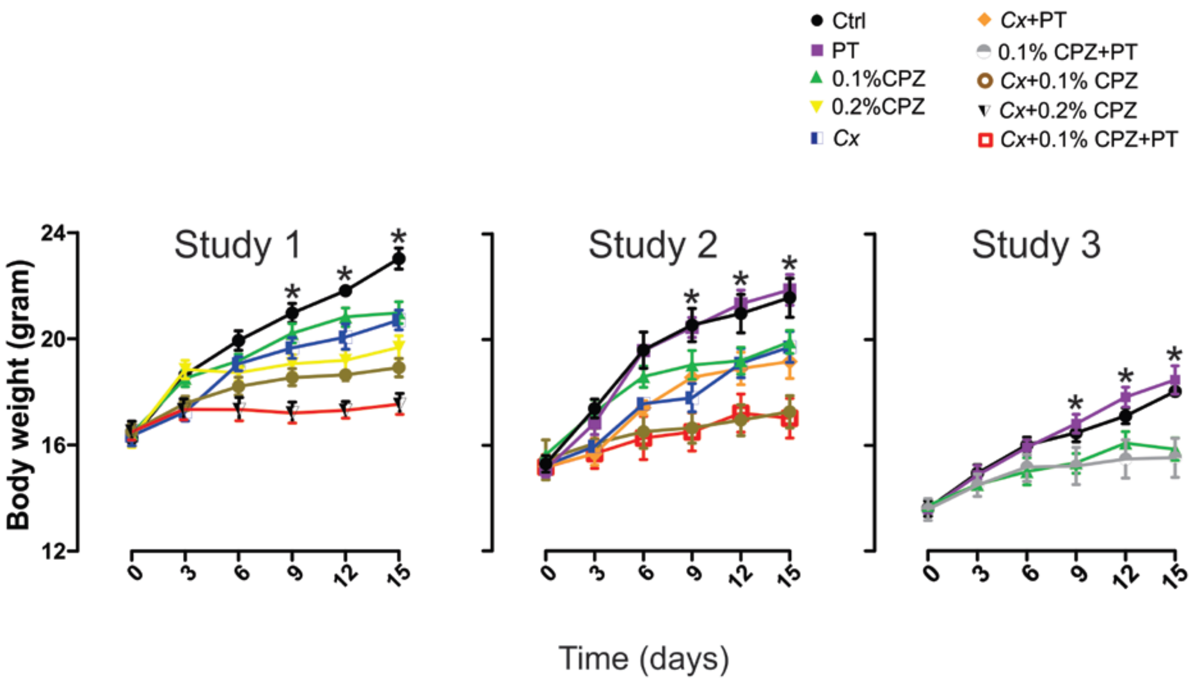

Supplementary FIGURE 2: Body weight data

Graphs (Studies 1, 2 and 3) show the total body weight differences among the experimental groups used in these studies. A slow gain of body weight in 0.1% and/or 0.2% CPZ-fed mice compared with corresponding *Cx* and Ctrl groups in Studies 1 and 2. *Cx* delayed the thriving of mice during the first three days in studies 1 and 2. In the Study 3, mice fed 0.1% CPZ showed a significantly slower weight gain compared with corresponding Ctrls (n=10 animals/group in study 1 and 3, n=5 in the Study 2); *significantly different from Ctrl (p<0.05).

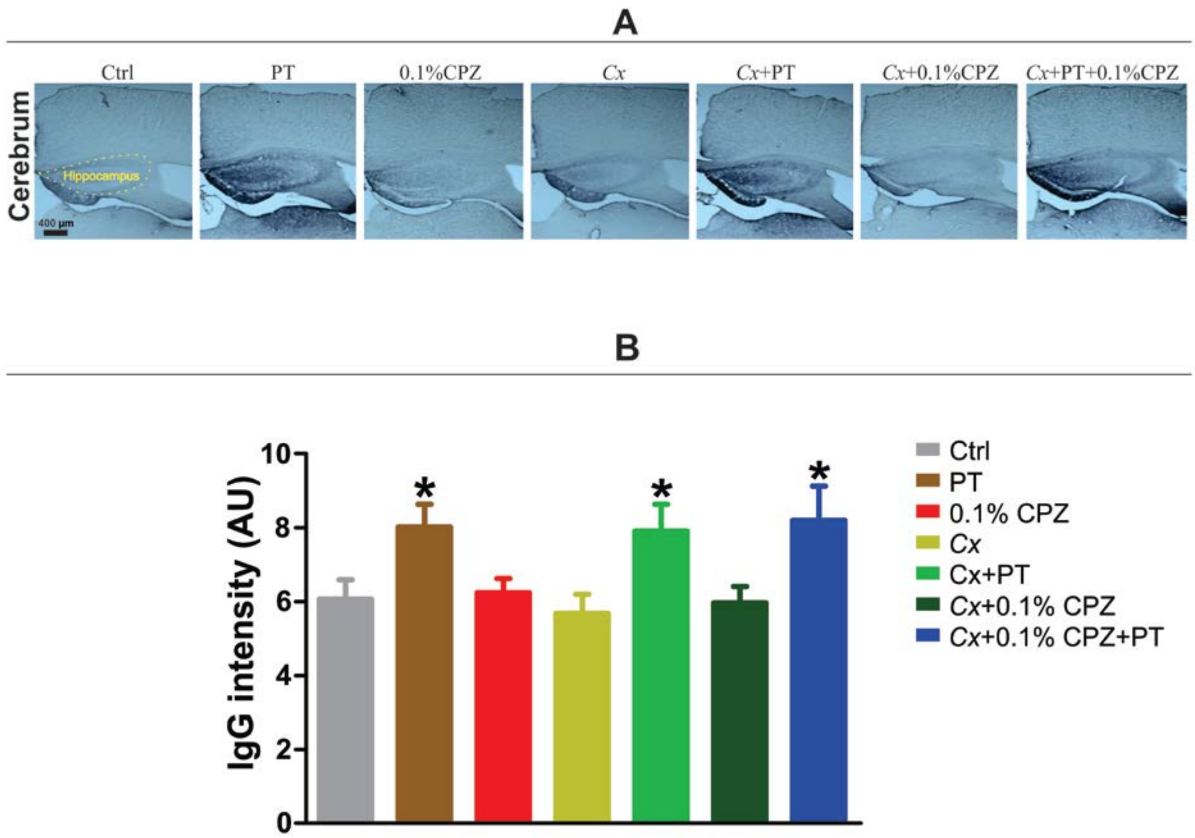

Supplementary FIGURE 3: Effect of PT on the permeability BBB

Representative images showing IgG staining in the brain sections following PT injection (A), and quantification (B) of IgG intensity. A significant (p<0.05) increase in the IgG colour intensity in the hippocampus region of PT injected groups were seen in Study 2. This effect indicates that immunoglobulin G traversed the BBB into the brain tissue (i.e. BBB was permeable)**.** n=3 mice/group, 10 sections/mouse, *significantly different from Ctrl (p<0.05).
